# Digital health usage patterns and digital health literacy: a deep dive into the Portuguese reality through a cross-sectional study

**DOI:** 10.3389/fpubh.2026.2009493

**Published:** 2026-10-30

**Authors:** Marta Estrela, Fátima Roque, Pedro Lopes Ferreira, Maria Teresa Herdeiro

**Affiliations:** 1 Department of Medical Sciences, iBiMED—Institute of Biomedicine, University of Aveiro, Aveiro, Portugal; 2 Department of Social, Political and Territorial Sciences, University of Aveiro, Aveiro, Portugal; 3 Health Sciences Research Center, University of Beira Interior (CICS-UBI), Covilhã, Portugal; 4 Centre for Health Studies and Research, University of Coimbra, Coimbra, Portugal; 5 Biotechnology Research, Innovation and Design for Health Products (BRIDGES), Polytechnic University of Guarda, Guarda, Portugal; 6 Centre for Innovative Biomedicine and Biotechnology (CIBB), University of Coimbra, Coimbra, Portugal

**Keywords:** cross-sectional, digital health, digital health literacy, health education, Portugal

## Abstract

**Objectives:**

With the increasingly widespread use of digital health tools and services, ensuring equitable access while making sure that citizens have the needed skillset to navigate the digital health world is essential. This study aimed to explore the digital health literacy (DHL) levels of the Portuguese population, more specifically those who already report using digital health tools, while assessing whether sociodemographic characteristics have an influence on DHL levels.

**Methods:**

A questionnaire containing sociodemographic questions and two DHL scales (HL-19-DIGI and TeHLI) was applied through a cross-sectional study. Multiple beta regression models were applied to assess the influence of sociodemographic characteristics on DHL.

**Results:**

1,047 people participated in this study. Digital health usage varied considerably among respondents, with websites and social media being the most used platforms. In terms of DHL, almost half of the respondents reported excellent HL-19-DIGI scores. However, there were differences between TeHLI’s functional, communicative, critical, and translational dimensions. Multiple beta regression models revealed that DHL levels consistently decrease as age increases, and that frequently using digital health tools and services is associated with higher DHL scores. In some cases, educational attainment level, residence area, and income also revealed influences in DHL levels. Exploratory analyses identified household income as a moderator across age, gender, and employment status.

**Conclusion:**

Our study provides an assessment of DHL within the Portuguese adult population who engage with digital health, identifying several factors hindering or facilitating effective digital health use. As we recognize these factors may exist alone or in combination with several other factors, our results reinforce the need for evidence-based and citizen-centered interventions.

## 1 Introduction

For the past decade, the healthcare landscape has been changing rapidly due to the expansion of digital health technologies (1, 2). Digital health technologies comprise telehealth, mobile health applications, wearable technologies such as smartwatches, electronic health records, and the vast universe of online health information (3). Thus, this expansion holds promising improvements in access to healthcare services, the quality of health services, and the efficiency of healthcare systems (4). However, achieving the potential that digital health brings is critically dependent on equitable access and effective utilization of digital health tools and services (5, 6). One of the main barriers that hinders a healthy expansion and adoption of digital health resides in the digital divide, which disproportionately affects vulnerable populations—including those with lower socioeconomic status, older adults, individuals in rural areas, and those with limited formal education (7). Therefore, without understanding the crucial role of digital health literacy (DHL) (8, 9), the effective implementation of digital health is delayed. Furthermore, a lack of attention to DHL risks widening existing health inequities and creating new ones.

Like digital health, DHL is an evolving concept that extends beyond basic computer skills; including the ability to effectively seek, find, understand, critically appraise, and apply health information from digital sources, and to use digital tools to manage one’s own health and participate in healthcare decisions (10, 11). Beyond the foundational definitions, DHL is considered a “superdeterminant” of health, involving the individual, institutions, and the society at large (12, 13). Thus, DHL consists of a complex skill set requiring not only technical competence but also cognitive and social capabilities (13). However, successful adoption and use of digital health is not simply a matter of DHL, as it is deeply connected with sociodemographic determinants such as age, gender, education level, income, employment status, and geographic location, that may facilitate or hinder the acquisition of this skillset (1, 12). Furthermore, digital health adoption is also influenced by context-specific intricacies relating to cultural norms, healthcare system infrastructures, language barriers, and the perceived trustworthiness of digital health sources (1, 14). Therefore, an individual’s willingness and ability to engage with digital health tools is heavily dependent on a complex network of factors. Moreover, not all influencing factors identified within a specific geographic location may necessarily exist in other regions (1, 15). This complexity highlights the need for nuanced research that considers both individual capabilities and the broader contextual factors influencing digital health engagement.

In Portugal, existing research suggests that approximately 30% of the adult population demonstrates limited health literacy skills (16), a challenge compounded by disparities in internet access and digital skills, with only 56% of the population possessing digital skills at a basic or above level (16). Considering that DHL comprises both computer skills and health literacy skills, a comprehensive study is urgently needed to better understand the reality in Portugal. One study exploring DHL levels at a nation-wide level found that over half of the population presented inadequate or problematic DHL levels (16). While large-scale population surveys assessing DHL through standardized psychometric batteries remain scarce, digital health usage, information-seeking behaviors, and digital inclusion have been actively investigated across various disciplines (17–20). This gap in literature underscores the importance of a broader investigation. Thus, this study aims to investigate the levels of digital health literacy amongst the Portuguese population who use at least one type of digital health tool, exploring the influence of key sociodemographic factors and contextual elements such as prior experience with technology and their ability to navigate and utilize digital health resources. Employing a cross-sectional study, this research aims to contribute to a nuanced understanding of the challenges and opportunities surrounding digital health adoption in Portugal.

## 2 Methods

### 2.1 Study setting

This cross-sectional study was conducted in Portugal, a Western European country with around 10.4 million inhabitants (21). Portugal is divided into nine regions [according to the Nomenclature of Territorial Units for Statistics (NUTS) II—Figure 1]: seven continental regions and two archipelago regions (22), presenting some regional variations in terms of population density (23), healthcare provision (24), demographic characteristics, and health status (25). Almost a quarter of the Portuguese population is aged 65 years old or older, whereas only 12.8% is aged 14 years old or less (26). Around 38.7% of the population has a lower-secondary education, being the country with the highest proportion of adults aged 15–64 years old with low education levels among European Union (EU) countries (26). Almost a third (32.7%) of the population has secondary or post-secondary education, while 28.6% have an higher education level, ranking 21st among EU countries (26).

**Figure 1:**
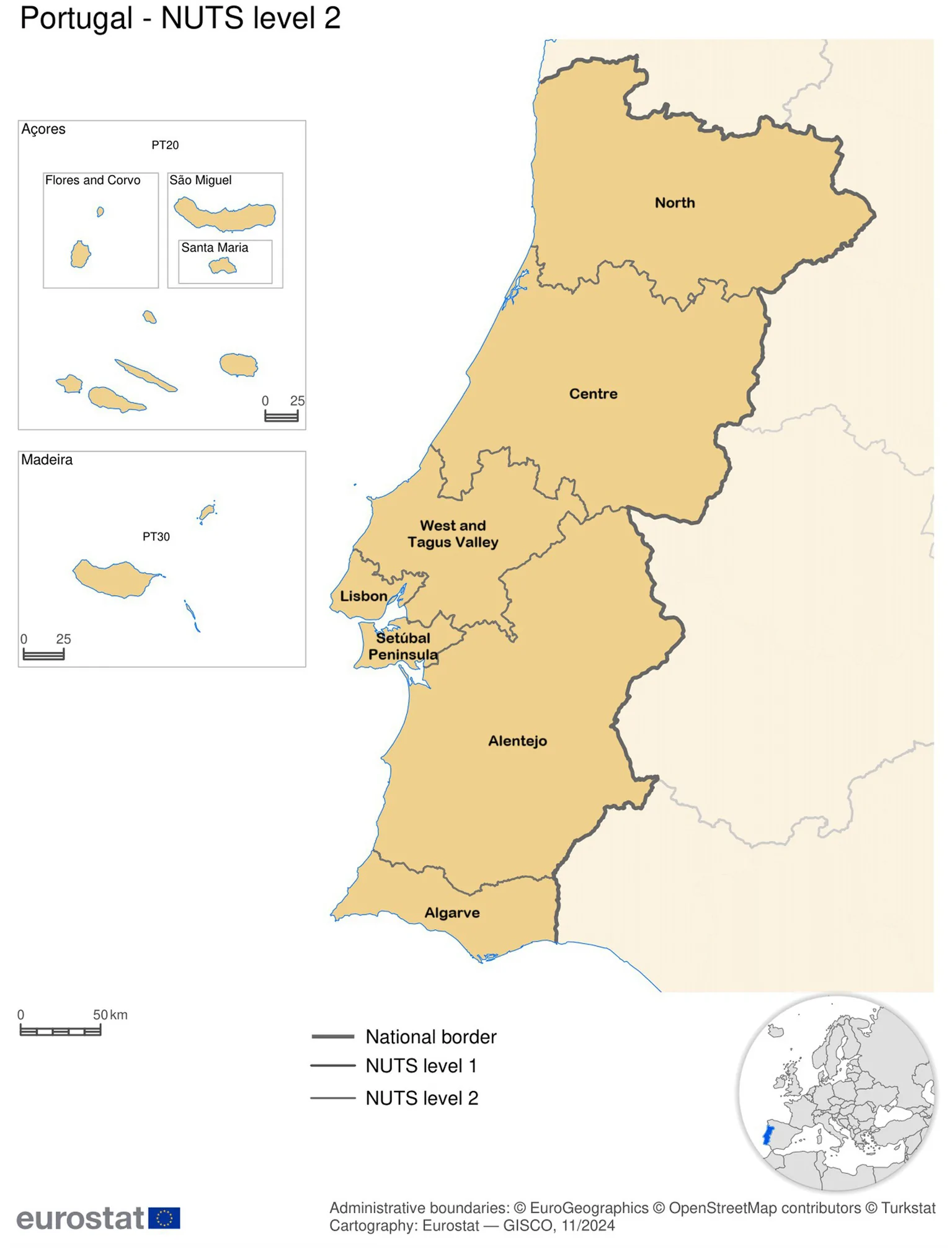
Portuguese NUTS II regions.

### 2.2 Questionnaire structure

The survey included questions on (1) demographic information (gender, age, nationality, marital status, residence area (i.e., geographical location), self-reported residence zone, based on population-size brackets provided in the questionnaire (i.e., urban, suburban, or rural), education level, family structure, employment status, and income [indexed to the national minimum wage at 14 months)], following national census standards (27); (2) the Portuguese version of the HLS19-DIGI scale (28, 29), a 8-item questionnaire measuring digital health literacy (HL-DIGI) with a 4-point Likert scale, followed by a 2-item questionnaire to measure interaction with digital devices (HL-DIGI-INT) with a 4-point Likert scale, and by a 6-item questionnaire to measure frequency of use of digital resources (HL-DIGI-DD) with a 7-item Likert scale; (3) a cross-culturally adapted and translated version of the Transactional eHealth Literacy Instrument (TeHLI) (30), comprising 18 items with a 5-point Likert scale. While both DHL scales essentially rely on self-reported perceptions on digital health use, the simultaneous use of both DHL scales is justified by their complementary conceptualizations of digital health literacy. The HL-19-DIGI essentially assesses perceived task difficulty across four core domains (accessing, understanding, appraising, and applying digital health information), providing a concise metric of information processing and credibility evaluation. On the other hand, the TeHLI operationalizes digital health literacy through four hierarchical dimensions: functional, communicative, critical, and translational, accounting for the interactive nature of contemporary digital healthcare.

### 2.3 Participant recruitment and inclusion criteria

A multi-faceted recruitment strategy was implemented, combining online and in-person approaches, from September 2024 to February 2025. Online recruitment targeted a diverse range of public and private institutions (senior universities and residences, universities and research hubs, schools, patients’ associations, private companies, vocational training and employment centres, and NGO’s), requesting to disseminate the study among their contact lists and via social media. Dissemination requests were re-sent once as a reminder to all institutions. In-person recruitment was conducted in public spaces, where people were approached to fill in the questionnaire after being informed of the study scope and providing their informed consent. No financial incentives were given to participants. The inclusion criteria were: (a) residing in Portugal; (b) being at least 18 years old; and (c) providing informed consent. Data were collected using a paper-based questionnaire, in case of in-person recruitment, and an online survey, hosted in the FormsUA platform, in the case of online recruitment. People who reported no use of any kind of digital resources were excluded from this study. Given the nation-wide scope of the study, a minimum sample size of 385 was also calculated based on a 95% confidence level and a 5% margin of error. This study was conducted as per the STROBE cross-sectional reporting guidelines (checklist available at the Supplementary material S1) and received favorable ethics approval from the Ethics and Deontology Council of the University of Aveiro (no. CED-46/2024), and ensured that all ethical standards and guidelines were adhered to throughout the research process.

### 2.4 Statistical analyses

Statistical analyses were performed using R (version 4.4.1), via RStudio 24.12.0, with the packages *dplyr*, *readxl*, *betareg*, *car*, *lmtest*, *ggplot2*, *broom*, *purrr*, *openxlsx*, and *writexl*. Descriptive statistics were calculated for all variables, including means, standard deviations, and frequencies. Responses with missing values were excluded from analyses, except for cases with missing data solely on the TeHLI scale. For participants with missing TeHLI data, those cases were excluded only from analyses specifically involving the TeHLI scale, allowing their data to be included in other analyses. HL-DIGI and HL-DIGI-INT scores were also calculated as the percentage of items with valid responses answered with “very easy” or “easy,” given that at least 80% of the items have valid responses (29, 31). For descriptive purposes, DHL levels were then categorized into “inadequate,” “problematic,” “adequate,” and “excellent,” following the official HLS19 Consortium guidelines. a standardized composite score (0–100) was calculated onto four standardized proficiency levels: Inadequate (scores 0–50), Problematic (>50–66.6), Sufficient (>66.6–83.3), and Excellent (>83.3–100) (28, 29). For regression analyses, averages were used for both HL-DIGI and TeHLI. Internal consistency of HL-DIGI and TeHLI were assessed using Cronbach’s alpha. To explore initial differences in means t-tests and one-way ANOVA were conducted to compare (1) TeHLI average scores, (2) digital health frequency of use, and (3) HL-DIGI and HL-DIGI-INT scores across sociodemographic groups. Univariate beta regression analyses were performed to assess the individual association of each potential predictor (sociodemographic variables and digital health frequency of use) with digital health literacy outcomes. Afterwards, multivariate beta regression analyses were conducted to assess whether sociodemographic variables and digital health frequency of use were associated with digital health literacy levels, while simultaneously adjusting for other variables in the model. Model assumption checks for normality, homoscedasticity, multicollinearity, and independence were performed to ensure the appropriateness of the analysis. Odds ratios and their respective 95% confidence intervals were calculated from the multivariable beta regression estimates to quantify the strength and direction of associations. To explore potential intersectional dynamics among sociodemographic determinants, two-way interaction terms were also evaluated using the Beta regression framework. An exploratory screening of all pairwise interactions among sociodemographic variables on TeHLI scores was conducted. To control for Type I error inflation across multiple comparisons, *p*-values from the interaction tests were adjusted using the Benjamini–Hochberg False Discovery Rate (FDR) procedure (32). Significance for all inferential tests was defined at a *p*-value ≤ 0.05.

## 3 Results

### 3.1 Sociodemographic characteristics

A total of 2,612 individuals initiated the online questionnaire, and 44 individuals completed the in-paper questionnaire. Of these, 1,582 were excluded due to incomplete responses, and 27 were excluded for reporting no use of digital health resources. This resulted in a final sample of 1,047 participants. Sociodemographic characteristics of the sample are available in Table 1.

**Table 1:** Sociodemographic characteristics of the sample.

Variable/Subgroup/n(%)	Variable	*N* (%)
Gender	Male	244 (23.30)
	Female	796 (76.03)
	Other/rather not say	7 (0.63)
Age	18–25	145 (13.85)
	26–35	167 (15.95)
	36–45	164 (15.66)
	46–55	215 (20.53)
	56–65	181 (17.28)
	66–75	136 (12.99)
	76+	39 (3.72)
Nationality	Portuguese	988 (94.36)
	Double nationality	33 (3.15)
	Foreign	26 (2.48)
Marital status	Single	362 (34.57)
	Married	543 (51.86)
	Separated/widowed	142 (13.56)
Residence area (NUTS II)	North	277 (26.46)
	Centre	277 (26.46)
	West and Tagus Valley	100 (9.55)
	Lisbon	166 (15.85)
	Setúbal Peninsula	91 (8.69)
	Alentejo	45 (4.30)
	Algarve	52 (4.96)
	Azores	14 (1.34)
	Madeira	25 (2.38)
Residence zone	Urban	682 (65.14)
	Suburban	211 (20.15)
	Rural	154 (14.71)
Education	Basic	22 (2.10)
	Secondary	49 (4.68)
	Post-secondary	157 (15.00)
	BSc	477 (45.56)
	MSc	271 (25.88)
	PhD	71 (6.78)
Family structure	Without children	723 (69.05)
	One adult	224 (30.98)
	One older adult	87 (12.03)
	Two or more adults	303 (41.91)
	Two or more adults, at least one older adult	109 (15.07)
	With children	324 (30.95)
	One adult with children	53 (16.36)
	Two or more adults, one child	129 (39.81)
	Two or more adults, two or more children	142 (43.83)
Employment status	Active	700 (66.86)
	Full-time	574 (54.82)
	Part-time	15 (1.43)
	Unemployed	65 (6.21)
	Self-employed	25 (2.39)
	Other	21 (2.01)
	Inactive	347 (33.14)
	Retired	156 (14.90)
	Student	191 (18.24)
Income (annual, gross)	Up to 9,310€	52 (5.72)
	9,310€–18,620€	260 (28.60)
	18,620€–27,930€	230 (25.30)
	27,930€–37,240€	154 (16.94)
	37,240€–55,860€	135 (14.85)
	Over 55,860€	78 (8.58)
Total	1,047	

### 3.2 Digital health usage patterns

Digital health usage varied considerably (Table 2). A substantial proportion of participants reported using websites (48.32%) and social media (41.58%) at least 1–3 times per week. Digital devices and mobile app usage were also common, with 38.45 and 36.61% using them at least 1–3 times per week, respectively. However, a notable proportion (11.86%) reported not interacting with health system digitally, while the majority (69.19%) did so less than once a week.

**Table 2:** Digital health usage patterns.

DH platform type	I do not use it	Less than once a week	1–3 days a week	4–6 days a week	Once a day	More than once a day	I do not know/rather not answer
Websites	71 (6.85)	461 (44.56)	245 (23.63)	74 (7.14)	50 (4.82)	132 (12.73)	4 (0.39)
Social media	248 (24.10)	342 (33.24)	152 (14.77)	59 (5.73)	80 (7.77)	137 (13.31)	11 (1.07)
Digital devices	354 (34.20)	263 (25.41)	132 (12.75)	58 (5.60)	96 (9.28)	112 (10.82)	20 (1.93)
Mobile apps	371 (35.71)	284 (27.33)	128 (13.32)	56 (5.39)	107 (10.30)	79 (7.60)	14 (1.35)
Digital interaction with health system	122 (11.86)	712 (69.19)	123 (11.95)	27 (2.62)	19 (1.85)	20 (1.94)	6 (0.58)
Other	473 (52.67)	297 (33.07)	48 (5.35)	17 (1.89)	14 (1.56)	21 (3.12)	21 (2.34)

### 3.3 Digital health literacy

#### 3.3.1 HL-DIGI and HL-DIGI-INT scores

Internal consistency for the HL-DIGI scale was high (Cronbach’s alpha = 0.86). When assessing the ease of judging the reliability of online health information (Table 3), most participants reported it was “easy” (56.35%) or “very easy” (32.66%). Yet, a substantial proportion found it “difficult” (8.31%) or “very difficult” (0.57%). Similar patterns were observed for understanding the information, while reporting slightly more difficulties in assessing the commercial interests of information available in the internet. Participants generally reported greater ease in finding information (84.81% “easy” or “very easy”) than in applying it to solve a health problem (57.26% “easy” or “very easy”). In turn, participants revealed more difficulties in using the information found to solve a health problem (37.91% “very difficult” or “difficult) and judging whether the information found is applicable to them (44.51% “very difficult” or “difficult”).

**Table 3:** HL-DIGI and HL-DIGI-INT distribution.

Variable/subgroup	Do not know/Rather not answer	Very difficult	Difficult	Easy	Very easy
When you search online for information on health, how easy or difficult is it for you… (HL-DIGI)					
1. To judge whether the information is reliable?	22 (2.10)	6 (0.57)	87 (8.31)	590 (56.35)	342 (32.66)
2. To judge whether the information is offered with commercial interests?	18 (1.72)	9 (0.86)	178 (17.00)	638 (60.93)	204 (19.48)
3. To understand the information?	17 (1.62)	5 (0.48)	103 (9.84)	656 (62.66)	266 (25.41)
4. To use the information to help solve a health problem?	5 (4.78)	63 (6.01)	334 (31.90)	458 (43.74)	142 (13.52)
5. To judge whether the information is applicable to you?	63 (6.02)	83 (7.93)	383 (36.58)	385 (36.77)	133 (12.70)
6. To find the exact information you are searching for?	41 (3.92)	14 (1.34)	104 (9.93)	588 (56.16)	300 (28.65)
7. To visit different websites to check whether they provide similar information about a topic?	34 (3.25)	27 (2.58)	243 (23.21)	570 (54.44)	173 (16.52)
8. To use the proper words or search query to find the information you are looking for?	77 (7.35)	64 (6.11)	356 (34.00)	447 (42.69)	103 (9.84)
When typing a health-related message on a digital device how easy or difficult is it for you to… (HL-DIGI-INT)					
1. Express your opinion, thoughts, or feelings, ask a question in writing on social media including online forums?	75 (7.16)	10 (0.96)	132 (12.61)	583 (55.68)	247 (23.59)
2. Clearly formulate your written message when communicating with a health provider?	185 (17.67)	26 (2.48)	170 (16.24)	490 (46.80)	176 (16.81)

The standardized composite score for HL-DIGI revealed that 17.09% of the participants reported inadequate levels of DHL, with a total score ranging from 0 to 50. A total of 22.15% reported problematic levels (50–66.6 score), while 13.84% revealed an adequate level (66.6–83.3 score) of digital health literacy, and 46.89% an excellent score (83.3–100 score). In line with HLS-DIGI, for the HL-DIGI-INT scale, 17.0% scored zero, corresponding to inadequate levels, while 23.11% scored 50 and 59.88% obtained the maximum score.

Regarding digital interaction skills (HL-DIGI-INT), most participants found it “easy” (55.68%) or “very easy” (23.59%) to express their opinions online, but slightly more challenging to clearly formulate written messages to healthcare providers (63.61% “easy” or “very easy”).

#### 3.3.2 TeHLI

The internal consistency for the TeHLI scale was excellent, presenting a Cronbach’s alpha of 0.95. The mean total TeHLI score was 3.50 (SD = 0.687), with average subscale scores as follows: functional: 3.726 (SD = 0.688); communicative: 2.942 (SD = 0.944); critical: 3.359 (SD = 0.837); and translational: 3.657 (SD = 0.794).

### 3.4 Associations between DHL and sociodemographic variables

Multiple beta regression models were employed to examine the relationships between sociodemographic characteristics, digital health utilization, and TeHLI, HL-DIGI, and HL-DIGI-INT (Table 4). Overall, the models demonstrated that DHL is not uniformly distributed across the Portuguese population, and that each subset of skills may vary differently across demographic groups. Full results are available on the Supplementary material S2.

**Table 4:** Betaregression results for each model: TeHLI and its subdimensions (functional, communicative, critical, and translational), HL-DIGI, and HL-DIGI-INT.

Variable/subgroup	TeHLI (*n* = 963)	Func. (*n* = 963)	Comm. (*n* = 963)	Crit. (*n* = 963)	Trans. (*n* = 963)	HL-DIGI (*n* = 1,047)	HL-DIGI-INT (*n* = 1,047)
(Intercept)	2.53^***^	2.53^***^	1.66’	2.58^***^	3.16^***^	1.19	1.40
Age *(ref: 18–25)*							
26–35	1.04	0.97	1.01	0.99	1.05	0.95	0.94
36–45	0.90	**0.78** ^*^	0.95	0.84	0.90	0.99	1.12
46–55	**0.73** ^**^	**0.76** ^*^	0.79’	**0.62** ^***^	**0.74** ^*^	0.89	0.99
56–65	**0.68** ^***^	**0.76** ^*^	0.79’	**0.57** ^***^	**0.52** ^***^	0.79’	0.77
66–75	**0.67** ^***^	0.87	0.84	**0.54** ^***^	**0.58** ^***^	**0.75** ^*^	1.05
76+	**0.63** ^**^	**0.68** ^*^	0.80	**0.50** ^***^	**0.52** ^**^	**0.57** ^**^	1.03
Gender *(ref: Male)*							
Female	0.98	**1.14** ^*^	0.94	0.98	0.86’	1.16*	1.21’
Nationality *(ref: Portuguese)*							
Double nationality	1.10	1.18	1.02	1.21	1.47*	1.14	0.80
Foreign	0.76’	**0.72** ^*^	0.73’	0.73’	0.81	0.73	0.74
Residence Area *(ref: North)*							
Centre	0.90	0.95	**0.85** ^*^	0.97	1.04	0.94	0.96
West and Tagus Valley	0.85’	0.98	**0.73** ^**^	0.98	0.98	0.94	0.92
Lisbon	0.95	0.91	0.86	0.93	**1.25** ^*^	1.18	0.90
Setúbal Peninsula	0.94	0.92	0.91	1.00	1.13	1.05	0.93
Alentejo	1.07	0.78’	1.10	1.04	1.05	1.01	1.25
Algarve	0.97	1.08	0.83	1.00	1.06	0.95	0.99
Azores	0.72	0.76	0.64’	0.82	0.58’	1.27	0.76
Madeira	1.06	1.01	0.81	**1.64** ^**^	1.56’	0.81	**0.55** ^*^
Residence zone *(ref: Urban)*							
Suburban	1.08	0.93	1.09	0.96	**1.20** ^*^	1.12	0.97
Rural	1.05	0.92	1.13	1.00	1.17	1.06	1.03
Education level *(ref: Basic)*							
Secondary	1.12	0.90	1.11	1.15	1.14	1.06	1.05
Tech specialization	0.99	0.95	1.02	0.92	1.08	1.49’	1.42
BSc	1.06	1.01	1.08	0.99	1.28	1.55’	1.60
MSc	1.26	1.19	1.32	1.24	1.51	**2.12** ^**^	1.48
PhD	1.38	1.44’	1.53’	1.28	1.45	1.73*	1.94’
Family structure *(ref: family w/no children)*							
With children	0.98	1.09	0.93	0.92	0.92	1.14’	0.94
Professional activity status *(ref: active)*							
Inactive	0.92	0.87’	0.89	0.83’	0.87	0.92	**0.65** ^**^
Income *(ref: below 9,310€/year)*							
9,310€–18,620€	**0.75** ^**^	0.93	**0.68** ^**^	0.86	**0.66** ^*^	**0.66** ^**^	**0.60** ^**^
18,620€–27,930€	0.89	1.17	0.80’	0.96	0.76’	**0.72** ^*^	**0.64** ^*^
27,930€–37,240€	0.94	1.17	0.82	1.17	0.76’	0.89	0.78
37,240€–55,860€	1.04	**1.36** ^*^	0.92	1.30’	0.87	0.98	0.75
Over 55,860€	1.00	**1.35** ^*^	0.90	1.19	0.80	0.99	0.75
Digital health device usage frequency	**1.16** ^***^	**1.08** ^**^	**1.22** ^***^	**1.14** ^***^	**1.22** ^***^	**1.26** ^***^	**1.23** ^***^
*Pseudo R* ^2^	0.14	0.08	0.10	0.11	0.11	0.11	0.08
Phi coefficient	9.52^***^	7.89^***^	5.14^***^	5.29^***^	4.40^***^	4.07^***^	1.27^***^

’*p* < 0.1, ^*^p < 0.05, ^**^p < 0.01, ^***^p < 0.001.

Estimates expressed as odds-ratios. Bolded values represent statistically significant value (*p* < 0.05).

Higher frequency of digital use was strongly associated with higher DHL scores in all models. For the first model, older age was associated with significantly lower TeHLI scores. The association between household income and digital health literacy revealed a non-linear pattern across dimensions (Table 4). Compared to the reference group (<9,310 €/year), participants in the lower-middle income tier (9,310€–18,620€) exhibited significantly lower scores across overall DHL and several sub-dimensions, with reductions persisting into the 18,620€–27,930€ bracket for HL-DIGI. Conversely, in the upper income tiers, the association reversed for the functional dimension, where participants earning 37,240€–55,860€ and over 55,860€ scored significantly higher than the baseline group. Scores in the remaining intermediate brackets did not differ significantly from the reference category.

Regarding TeHLI’s functional sub-dimension, age, female gender, nationality, and income were significantly associated with differences in functional scores. For the communicative subscale, residence area and income were observed as influencing factors. Critical skills presented significant differences among older individuals, along with residence area. Age, residence area and residence zone, and income, were identified as influencing factors of translational skills.

To explore potential intersectional dynamics among sociodemographic determinants, all 55 pairwise two-way interactions predicting TeHLI scores were systematically evaluated using Beta regression Likelihood Ratio Tests (Supplementary file S3). Across all evaluated pairs, three interactions survived multiple-testing correction: age-income, gender-income, and active labor status-income. While higher income provided protective literacy returns in middle-aged cohorts (36–45 years), it did not offset the digital penalty among adults aged 76+, who exhibited persistent negative coefficients across higher income tiers. Across all income tiers above the minimum wage baseline (< 9,310 €), female participants exhibited significantly negative interaction coefficients, showing that the positive returns of household income on transactional literacy are significantly steeper for men than for women. Inactive participants experienced consistently negative interaction terms across higher income intervals, suggesting that the DHL benefits of household wealth are attenuated without active workplace or educational engagement.

In terms of digital health literacy measured through HLS-DIGI, only those older than 65 present lower DHL levels. Gender and income are also associated with differences in DHL scores. Unlike TeHLI and its subscales, higher education was associated with higher scores. For the last model, depicting the digital interaction with health system (HL-DIGI-INT), individuals from Madeira and those actively not employed had significantly lower levels of digital interaction with the health system. Lower income was associated with lower interaction skills.

## 4 Discussion

This study provides a comprehensive assessment of DHL within the Portuguese adult population who engage with digital health. Our findings reveal that DHL is comprised of a varied set of skills, being influenced by different sociodemographic factors and digital usage patterns. Despite regular engagement with digital technologies of most of the inquired populations, significant disparities exist in DHL levels, particularly linked to age, education, income, and geographic location.

A negative association between age and both TeHLI scores and HL-DIGI was observed, highlighting a persistent digital divide, consistent with our previously published meta-analysis (1). Older adults consistently demonstrated lower levels of digital health literacy in most of TeHLI dimensions, which can potentially reflect their challenges in adapting to new technologies, limited exposure, or cognitive and physical changes associated with aging. As we recognize these factors may exist alone or in combination with several other factors, our results reinforce the need for tailored and evidence-based interventions (33, 34) designed to address the specific needs and barriers experienced by older adults, focusing on building confidence and providing accessible training, possibly even taking advantage of already existing initiatives or infrastructure at a community level, such as local parishes and intergenerational programmes (35).

We also observed a positive association between higher educational levels (particularly master’s and PhD levels) and DHL, reinforcing that education plays a crucial role in digital health literacy and critical appraisal skills. Thus, our findings suggest that integrating digital health literacy into educational curricula at all levels could be a valuable long-term strategy, along with investments into educational programmes, public health campaigns, and lifelong learning programmes. This could potentially be achieved through employment and vocational centres, or by offering courses to improve DHL while raising awareness to available, evidence-based, digital health sources and services.

Income emerged as a significant predictor of both overall DHL and specific dimensions, with lower income levels consistently associated with reduced DHL. This may be related to lower access to internet, digital equipments, and health services (36, 37). Rather than a simple linear gradient, the relationship between household income and digital health literacy displayed a distinct non-linear pattern. Participants in the lowest income bracket (<9,310 €/year) exhibited higher scores than those in the lower-middle income tier (9,310€–18,620€), who recorded the lowest literacy estimates across several dimensions. A cross-tabulation of income against employment status clarifies this non-linear dynamic: the lowest income tier (<9,310 €/year) was disproportionately composed of university students and retirees, artificially elevating the baseline average. In contrast, among full-time employed participants, TeHLI scores exhibited a consistent, positive upward gradient with income, rising steadily from 3.12 in the lowest tier to 3.71 in the 37,240–55,860 € bracket.

Moreover, this financial gradient was evident not only in broader DHL, but specifically in functional competencies in TeHLI. This may be explained by higher-speed personal connectivity, updated devices, and private digital access, disproportionately concentrated in higher-income households, enabling digital health engagement such as secure messaging with clinicians, navigating patient portals to review lab results, and managing e-prescriptions (38). This structural inequality is further reinforced by our exploratory pairwise interaction analysis, which revealed that income serves as the primary moderating axis of DHL, indicating that daily functional immersion in digital workflows (via modern workplaces or higher education) is essential to translate economic capital into actionable digital health competence. This finding is particularly concerning, as it suggests that digital health may exacerbate existing inequities, creating a two-tiered system where those with fewer resources are further disadvantaged (39). Addressing this requires not only affordable access to technology and internet but also context-sensitive digital health resources. Interestingly, some regional variations were observed, with lower scores in the Centre and West and Tagus Valley regions, suggesting that access to resources and infrastructure may also play a role (40). The higher scores observed in Madeira warrant further investigation to identify potential best practices.

The strong positive association between frequency of digital use and DHL across all models suggests a reciprocal relationship: increased digital engagement fosters greater digital literacy, and vice versa. This reinforces the importance of promoting digital inclusion and providing opportunities for individuals to develop their digital skills through regular practice and exposure. The finding that inactive individuals (e.g., retired or students) were less likely to interact digitally with the health system suggests that employment status may also be a relevant factor, potentially reflecting differences in access to workplace-based digital health resources (41).

A nuanced examination of the TeHLI subscales revealed that while overall TeHLI was influenced by a range of factors, specific dimensions were more strongly associated with particular demographics. For example, communicative skills were lower in the Centre and West and Tagus Valley regions, while critical appraisal skills were higher in Madeira. Translational skills were higher for those with double nationality, from Lisbon, or living in suburban areas. These findings highlight the importance of tailoring interventions to address the specific needs of different communities.

## 5 Limitations and strengths

This study has several strengths, including its relatively large sample size, the use of validated instruments (HLS19-DIGI and TeHLI), and the comprehensive assessment of both DHL and digital health usage patterns. However, this study also has some limitations. The recruitment process, while aiming for broad reach, likely introduced selection bias. The use of online recruitment via social media and email, coupled with dissemination requests to specific institutions, may have excluded individuals with limited internet access, lower digital literacy skills, or those not affiliated with the targeted organizations. More specifically, individuals with lower levels of formal education, who are often less digitally engaged, may have been underrepresented in the sample, along with those from less densely populated areas. While in-person recruitment was employed to mitigate this, it was likely insufficient to fully counteract the bias inherent in the online recruitment strategy, as it refers to less than 5% of the observations. Consequently, the generalizability of the findings to the entire Portuguese adult population should be interpreted with caution. Another limitation of this study is the marked gender imbalance of the sample, with female respondents accounting for 76.0% of participants. This asymmetry stems from self-selection biases inherent to voluntary web-based health research, where male participation is often substantially lower than expected and women engage more frequently in online health information seeking as primary health managers within households (42–44). While our findings provide valuable insight into the demographic group most actively navigating digital health tools in Portugal, the underrepresentation of men limits the direct generalizability of these results to the broader, gender-balanced adult population. Future studies should employ gender-stratified quota sampling to ensure balanced population representation across all age and socioeconomic cohorts. As this sample is not representative of the Portuguese population, particularly regarding educational attainment and geographical distribution, it is possible that the observed levels of DHL are overestimated, as individuals who chose to participate in the study may have been more digitally literate and motivated to engage with digital health resources. Furthermore, the cross-sectional design limits our ability to establish causal relationships. Finally, the reliance on self-reported data introduces the potential for recall bias and social desirability bias (45). Participants may have overestimated their digital skills or provided responses that they believed were socially acceptable.

## 6 Conclusion

The findings of this study have important implications for policymakers, healthcare providers, and digital health developers. Ultimately, our findings are helpful for the development of targeted interventions to promote equitable access to and effective utilization of digital health resources within the Portuguese healthcare system. Investing in digital literacy training programs, particularly for older adults and individuals with lower socioeconomic status, is crucial for promoting equitable access to digital health services. Moreover, developing culturally sensitive and linguistically appropriate digital health resources is essential for reaching diverse populations.

Future research should focus on exploring the specific barriers and facilitators to digital health adoption among underrepresented groups, to gain a deeper understanding of their experiences and perspectives, especially those from underserved populations with lower access to digital tools and educational attainment. Moreover, longitudinal studies are needed to explore the long-term impact of digital health interventions on health outcomes and to identify the most effective strategies for promoting digital health accessible for all. Finally, research should explore the role of healthcare providers and organizations at a community level in promoting digital health literacy and supporting patients and citizens in navigating the digital health landscape.

## Data Availability

The raw data supporting the conclusions of this article will be made available by the authors, without undue reservation.
